# Case of Blindness from Effusion of Blood in the Chambers of the Eye, from Suppressed Catamenia, Cured by Electricity

**Published:** 1801-01

**Authors:** 

**Affiliations:** Surgeon, of Brentford


### *Case of Blindness from Effusion of Blood in the Chambers of the Eye, from suppressed Catamenia, cured by Electricity; by Mr.* Ricards, *Surgeon, of Brentford.*

To Dr. BRADLEY.

Sir,

The following cafe being fuch, I think, as deferves the attention of the public, I beg leave, through the medium of your Journal, to make it known.

Mifs ——, aged 17 years, after a troublefome head-ache, which continued feveral days, became nearly blind of one eye, fo much fo that fhe could only diftinguifh light from darknefs; and every object which fhe attempted to view, appeared of a reddifh hue. Her friends, much alarmed at this fudden acceffion of fo terrible a malady, as the eye had to them a moft frightful appearance, immediately made application to the moft eminent of the faculty, particularly to fuch as made the difeafes of the eye the principal part of their practice. By accurate examination, it appeared that the chambers of the eye were filled by an opake, dark coloured fluid, inftead of the tranfparent aqueous humor, and the whole eye appeared fuffufed with blood. Confidering the difeafe as local, fuch remedies were directed as are generally employed to diminifh local plethora, and to promote the abforption of the effufed fluid. Leeches were applied to the temple, cathartics, blifters, &c. and fhe continued to follow thefe directions, and to purfue this plan of treatment for feveral months; but as no amendment was perceptible, fhe became weary of a courfe from which fo little benefit was derived; at length, fhe was advifed by a friend to to try the effects of electricity, who requested her to wait upon some practical electrician, and desire him to pass a stream of the aura from a wooden point into the eye daily, which she did accordingly.

The nature of the case being very fully inquired into, (for, being consulted, I never chuse to make use of any means without knowing a reason why) and the progress of the disease, and the methods hitherto used, being very satisfactorily explained by the young lady's mother, it appeared that the blindness was more considerable at sometimes than at others, previous to which there was generally a considerable head-ache; and that these occurrences for the most part took place about every third or fourth week; and that the disease had from the beginning been attended with obstructed catamenia, which circumstance had been very much overlooked by the oculists who had been previously consulted; but though no menstrual evacuation had taken place, the usual fore-running symptoms had generally occurred. If we stop a moment to theorize upon the disease, it would seem, that Nature had made an effort to perform her regular office; but from some cause of inactivity in the uterine system, the proper catamenial secretion had not taken place, in consequence of which the superabundant blood had fallen upon some other part; the vessels, in this instance, which secrete the aqueous humor had become enlarged and ruptured, and pure blood, or blood mixed with the aqueous humor, had been poured into the chambers of the eye. The peculiarity of this case is in the part on which Nature has directed her efforts; we frequently see her pouring out her periodical discharges by the lungs, by the vessels of the Schneiderian membrane, or by ulcers in different parts of the body. In such cases, it appears to me that we should not apply our remedies to the eye, the nose, the lungs, or the ulcer, but to the uterus; for if this organ can be excited to a proper secretion, the evacuations from the other parts will soon cease; the sequel of the present case will prove this to be true, in this instance at least.

I consider that the remedies employed to produce this effect on the uterus should be made use of about the time when the usual symptoms, which precede menstruation begin to appear; therefore, in order to divert the patient, but without expecting any real benefit from such a mode of application in a case of this kind, she had, daily, streams of electric aura passed into the eye, and likewise small shocks through the pelvis, waiting till symptoms indicated an effort of Nature to perform her proper office.

When such symptoms began to appear, as pain in the head and and loins, tenfion of the breafts, &c. the fhocks were greatly increafed both in number and ftrength, without informing her of any intention of making fuch an increafe of power; in a day or two the catamenial evacuation took place in a proper manner, and the eye was vifibly clearer; the electricity was omitted till the next periodical return of fymptoms, when it was repeated; the catamenia again appeared, and the eye very fpeedily recovered its original tranfparency, nor did any return of obftruction take place.

I confider this cafe as one on which much may be faid, relative to the good effects of electricity in uterine cafes; I would not be underftood from hence to recommend electricity in every cafe in which obftructed menftruation is a fymptom; in fome fuch cafes I am well aware that its ufe would be injurious, rather than falutary. I would diftinguifh thofe cafes in which amenorrhœa is a caufe, from thofe in which it is an effect of the difeafe. Chlorofis, I confider as a difeafe of the latter kind; the fymptoms, here, all indicate a paucity of blood, and Nature, in this cafe, acting as a judicious phyfician, prevents the lofs of that fluid, whofe deficiency is the very effence of the difeafe, by reftraining this cuftomary evacuation; it would therefore, I think, be highly improper to endeavour to force fuch an evacuation by any remedy which may be confidered as emenagogue, of which I certainly confider electricity to be the moft active; neverthelefs, I know that it is the practice of fome gentlemen of the profeffion to advife venæfection, almoft invariably, in this difeafe. But where there is fuppreffed menftruation from any accidental caufe, where no fymptoms of paucity of blood exifts, or where Nature points to fome other emunctory, or ruptures veffels in remote parts of the body, here the re-production of the catamenia muft be our chief concern; and to effect this, from repeated experience I can affirm, and I know it likewife to have been the refult of the practice of others, electricity will effect more than perhaps any other remedy; and if ufed in combination with other remedies, will greatly affift them in their curative intention. I have the honour to be,

Sir,

Your moft obedient fervant,

*Brentford, Dec. 14, 1800.* JASPER RICARDS.

